# Sarcopenic obesity and health outcomes: An umbrella review of systematic reviews with meta‐analysis

**DOI:** 10.1002/jcsm.13502

**Published:** 2024-06-19

**Authors:** Nicola Veronese, Francesco Saverio Ragusa, Francesco Pegreffi, Ligia J. Dominguez, Mario Barbagallo, Michela Zanetti, Emanuele Cereda

**Affiliations:** ^1^ Department of Health Promotion, Mother and Child Care, Internal Medicine and Medical Specialties University of Palermo Palermo Italy; ^2^ Dipartimento di Scienze per la Qualità della Vita‐QUVI Università di Bologna Rimini Italy; ^3^ Faculty of Medicine and Surgery “Kore” University of Enna Enna Italy; ^4^ Geriatric Clinic Maggiore Hospital of Trieste, Azienda Sanitaria Universitaria Integrata Giuliano Isontina Trieste Italy; ^5^ Department of Medical, Surgical and Health Sciences University of Trieste Trieste Italy; ^6^ Clinical Nutrition and Dietetics Unit Fondazione IRCCS Policlinico San Matteo Pavia Italy

**Keywords:** meta‐analysis, obesity, sarcopenia, sarcopenic obesity, umbrella review

## Abstract

Many studies support the idea that sarcopenic obesity (SO) could be considered a potential risk factor for negative health outcomes. These results have been inconsistent, and no umbrella reviews exist regarding this topic. Several databases until November 2023 were searched for systematic reviews with meta‐analysis of observational studies (cross‐sectional, case–control and prospective). For each association, random‐effects summary effect sizes with correspondent 95% confidence intervals (CIs) were evaluated using the GRADE tool. Among the 213 papers initially screened, nine systematic reviews with meta‐analysis were included, for a total of 384 710 participants. In cross‐sectional and case–control studies, 30 different outcomes were analysed, and 18 were statistically significant. In any population addressed in cross‐sectional and case–control studies, compared with non‐SO, SO increased the prevalence of cognitive impairment (*k* = 3; odds ratio [OR] = 3.46; 95% CI: 2.24–5.32; high certainty of evidence), coronary artery disease (*k* = 2; OR = 2.48; 95% CI: 1.85–3.31) and dyslipidaemia (*k* = 3; OR = 2.50; 95% CI: 1.51–4.15). When compared with sarcopenia or obesity, the results were conflicting. In prospective studies, the association between SO—compared with non‐SO—and other negative outcomes was supported by low/very low certainty of evidence and limited to a few conditions. Besides, no comparison with sarcopenia or obesity was provided. Finally, only a few studies have considered muscle function/physical performance in the diagnostic workup. SO could be considered a risk factor only for a few conditions, with the literature mainly based on cross‐sectional and case–control studies. Future studies with clear definitions of SO are needed for quantifying the importance of SO—particularly when compared with the presence of only sarcopenia or obesity—and the weight of muscle function/physical performance in its definition.

## Introduction

Sarcopenia, defined as the pathological loss of muscle mass and function, is very common in older people.^1^ Its prevalence ranges from 10% to 40% in community‐dwelling older adults. It has gained increased recognition as an important condition in older adults, which has helped to progress and expand the related field of research.^2^ Sarcopenia is associated with several adverse health‐related outcomes in older people, and its associations with mortality, disability and falls are supported by highly suggestive evidence.^3^

Similarly, the prevalence of obesity, defined as a pathological amount of fat mass,^4^ continues to rise over time. Recent data reported that adults over the age of 60 have obesity rates exceeding 37.5% in males and 39.4% in females in the United States.^5^ In an ageing population, this obesity epidemic represents a mounting financial concern with regard to healthcare resources.^6^ High adiposity levels are associated with increased cardiovascular disease (CVD) risk, despite divergent evidence gradients. Adiposity was a traditional risk factor for CVD,^7^ independently from other cardiovascular risk factors.^8^

There are complex interactions between sarcopenia and obesity, with multiple factors implicated in the maintenance of fat and muscle mass.^9^ The co‐existence of excess adiposity and low muscle function and mass is defined as sarcopenic obesity (SO), a condition increasingly recognized for its clinical and functional features that could negatively influence important patient‐centred outcomes.^10^ Some recent systematic reviews have reported that SO is a highly present condition over the lifespan. For example, in children and adolescents, the prevalence of SO could range from 5.66% to 69.7% in girls and between 7.2% and 81.3% in boys.^11^ In cancer, the prevalence of SO ranged from 6% to 40%.^12^ Finally, among older people, the prevalence of SO is ~1 person over 10.^9^

As SO is a highly prevalent condition, knowing and weighting its impact as a risk factor for other conditions is a public health priority. Given this background, the aim of this work is to assess—through an umbrella review^13^ (i.e., a review of other published meta‐analyses)—the strength and credibility of the evidence derived from systematic reviews with meta‐analyses on SO as a potential risk factor for health outcomes in observational studies. The importance of this issue is clearly emphasized by the heterogeneity in definition and diagnostic criteria across studies, which can clearly hamper the objective evaluation of this clinical entity—in terms of prevalence and prognostic impact—as well as the possibility of developing prevention and treatment strategies in different healthcare and disease settings.^10^

## Methods

### Protocol and registration

This systematic review was conducted following the recommendations of the Cochrane handbook for systematic literature reviews to carry out the screening and selection of studies and reported according to the updated 2020 Preferred Reporting Items for Systematic Review and Meta‐Analysis (PRISMA) guidelines.^14^, ^15^ The protocol is freely available at https://osf.io/rb9qt/.

### PICO question and eligibility criteria

Following the PICOS (participants, intervention, control, outcomes, study design) question, we included

- participants: any;
- intervention: presence of SO using any definition;
- controls: non‐SO. The controls were then divided into non‐SO (this group may include people with obesity, sarcopenia, normal weight and underweight), only obesity and only sarcopenia;
- outcomes: all health outcomes; and
- study design: systematic reviews and meta‐analyses of observational studies.

We excluded the following studies: (i) meta‐analyses of intervention studies in people affected by SO; (ii) data not meta‐analysable (e.g., systematic reviews without meta‐analysis); and (iii) meta‐analyses including only one observational study.

### Information sources and search strategies

For this umbrella review, several relevant bibliographic databases were comprehensively searched, including Medline (via Ovid), Embase and Web of Science, from database inception up to 17 November 2023.

The following search was used in PubMed: ((((((((((((((((((((‘Muscular Atrophy’[Mesh]) OR (Atrophies, Muscular[Title/Abstract])) OR (Atrophy, Muscular[Title/Abstract])) OR (Muscular Atrophies[Title/Abstract])) OR (Atrophy, Muscle[Title/Abstract])) OR (Atrophies, Muscle[Title/Abstract])) OR (Muscle Atrophies[Title/Abstract])) OR (Muscle Atrophy[Title/Abstract])) OR (Neurogenic Muscular Atrophy[Title/Abstract])) OR (Atrophies, Neurogenic Muscular[Title/Abstract])) OR (Atrophy, Neurogenic Muscular[Title/Abstract])) OR (Muscular Atrophies, Neurogenic[Title/Abstract])) OR (Muscular Atrophy, Neurogenic[Title/Abstract])) OR (Neurogenic Muscular Atrophies[Title/Abstract])) OR (Neurotrophic Muscular Atrophy[Title/Abstract])) OR (Atrophies, Neurotrophic Muscular[Title/Abstract])) OR (Atrophy, Neurotrophic Muscular[Title/Abstract])) OR (Muscular Atrophies, Neurotrophic[Title/Abstract])) OR (Muscular Atrophy, Neurotrophic[Title/Abstract])) OR (Neurotrophic Muscular Atrophies[Title/Abstract])) OR (((((Age related muscle loss[Title/Abstract]) OR (Age‐related muscle loss[Title/Abstract])) OR (Muscle insufficiency[Title/Abstract])) OR (Muscle depletion[Title/Abstract])) OR (Skeletal muscle depletion[Title/Abstract])) AND (((‘Obesity’[Mesh]) OR (Obese[Title/Abstract])) OR (Overweight[Title/Abstract])) AND (‘meta‐analysis’[ptyp] OR metaanaly*[tiab] OR ‘meta‐analy*’[tiab] OR ‘systematic review’[ptyp] OR ‘systematic review’[Title/Abstract]). The search was then adapted to the other databases.

### Study selection

The selections were independently carried out by two review authors (N. V. and F. P.), with consensus meetings to discuss the studies for which divergent selection decisions were made by the two review authors. A third senior member of the review team (E. C.) was involved, if necessary. The study selection process involved, first, a selection based on title and/or abstracts and, then, a selection of studies retrieved from this first step based on the full‐text manuscripts. The freely accessible software Rayyan was used for the title/abstract screening.^16^

### Data collection and data items

From the eligible full‐text articles, we extracted the following: first author name and affiliation, year of publication, journal name and title of the manuscript; data on the characteristics of the population considered for individual observational studies (e.g., sample size, mean age, location, gender, population/condition/setting, etc.); the study design; the type of control group (non‐SO [no obesity + no sarcopenia], only obesity and only sarcopenia); and the diagnostic criteria used for SO and health outcomes. The data regarding estimates were extracted at the single study level and categorized into risk ratio (RR), odds ratio (OR), hazard ratio (HR) and mean difference (MD). These data were collected using a standardized Excel data extraction form. The data extraction was led out by one review author (N. V.) and systematically double‐checked by a second review author (F. S. R.). Errors found in extraction by the second review author were corrected during a consensus meeting by both authors and a third member of the team (E. C.).

### Assessment of risk of bias

One author (F. P.) rated the methodological quality of the included systematic reviews using ‘A MeaSurement Tool to Assess systematic Reviews 2 (AMSTAR 2)’,^17^ which ranks the quality of a meta‐analysis in one of four categories ranging from ‘critically low’ to ‘high’ according to 16 predefined items. Another author (N. V.) double‐checked this evaluation.

### Data synthesis and grading of the evidence

The data analysis was conducted using STATA 14.0. For each meta‐analysis, we estimated the common effect size and its 95% confidence interval (CI) under the assumption of a random‐effects model.^18^ Heterogeneity was estimated using the *I* ^2^ statistics: Values of 50% or greater are indicative of high heterogeneity, while values above 75% suggest very high heterogeneity.^19^ Publication bias was assessed using the test proposed by Egger and co‐workers.^20^

The evidence from meta‐analyses was evaluated using the GRADE (Grading of Recommendations, Assessment, Development and Evaluation) assessment. The GRADE framework takes into account several important domains for the judgement of the certainty of the evidence, including study design, risk of bias, inconsistency, indirectness, imprecision and other aspects such as publication bias or a strong association between the exposure (i.e., SO) and the outcomes of interest.^21^ *Table* *S1* reports the criteria used, for each domain, for doing the GRADE. The certainty of the evidence was then evaluated as very low (the true effect is probably markedly different from the estimated effect), low (the true effect might be markedly different from the estimated effect), moderate (the true effect is probably close to the estimated effect) or high (there is a lot of confidence that the true effect is similar to the estimated effect).^21^ The results of the data analysis were imported into the GRADEpro Guideline Development Tool (McMaster University, 2015; developed by Evidence Prime, Inc.).

### Ethical issues

This study did not involve patients or any human or animal material and therefore does not imply any ethical issue.

## Results

### Literature search

**Figure 1:**
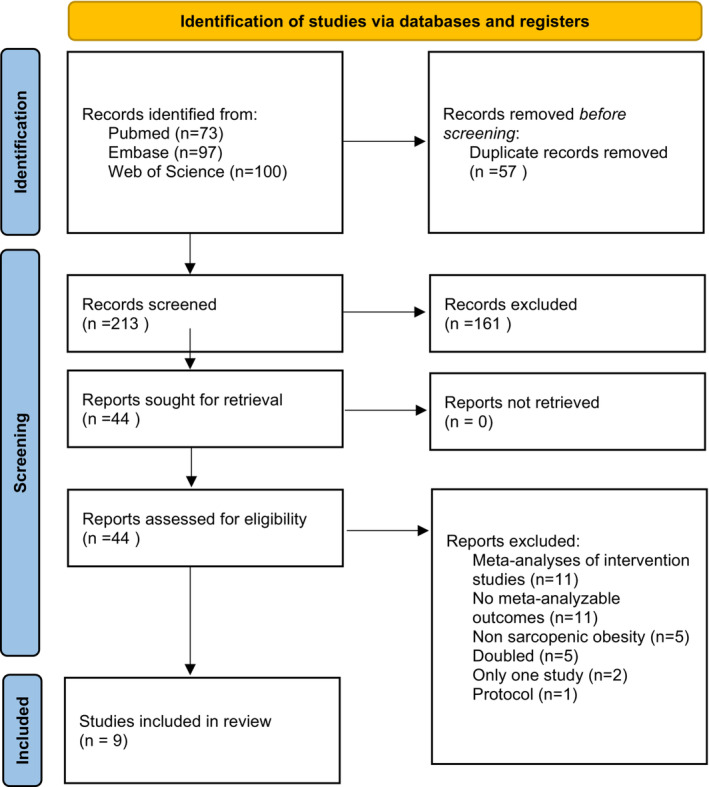
PRISMA flow.

As shown in *Figure* *1*, among the 213 papers initially screened, we evaluated 44 full texts. After excluding 35 full texts, mainly based on the fact that they were meta‐analyses of intervention studies or data that were not meta‐analysable, nine systematic reviews with meta‐analysis were included.^9^, ^22^, ^23^, ^24^, ^25^, ^26^, ^27^, ^28^, ^29^ The list of excluded references is reported in *Table* *S2*.

### Main findings of the umbrella review

The nine meta‐analyses included approximately 384 710 participants. *Tables* *1* and *2* report the main findings of the umbrella review in cross‐sectional and case–control studies and in cohort studies, respectively.

**Table 1:** GRADE profile of the associations between sarcopenic obesity (SO) and the outcomes of interest in cross‐sectional and case–control studies

Outcome	Population	Number of studies	Control group	Risk of bias	Inconsistency	Indirectness	Imprecision	Other considerations	Estimate (95% CI)	Overall certainty of evidence
Prevalence of cognitive impairment	Any	3	Non‐SO	Not serious	Not serious	Not serious	Not serious	Very strong association	OR 3.46 (2.24 to 5.32)	High
Prevalence of coronary artery disease	Any	2	Non‐SO	Not serious	Not serious	Not serious	Not serious	Strong association	OR 2.48 (1.85 to 3.31)	Moderate
Prevalence of dyslipidaemia	Any	3	Non‐SO	Not serious	Not serious	Not serious	Not serious	Strong association	OR 2.50 (1.51 to 4.15)	Moderate
Prevalence of cancer	Any	6	Non‐SO	Not serious	Not serious	Not serious	Not serious	None	OR 1.07 (0.90 to 1.28)	Low
Prevalence of fractures	Any	3	Non‐SO	Not serious	Not serious	Not serious	Not serious	None	OR 0.89 (0.44 to 1.78)	Low
Prevalence of functional limitations	Any	8	Non‐SO	Not serious	Not serious	Not serious	Not serious	Strong association	OR 2.92 (2.12 to 4.02)	Low
Prevalence of lung diseases	Any	4	Non‐SO	Not serious	Not serious	Not serious	Not serious	None	OR 1.62 (1.07 to 2.47)	Low
Prevalence of stroke	Any	8	Non‐SO	Not serious	Not serious	Not serious	Not serious	None	OR 1.82 (1.47 to 2.26)	Low
Prevalence of arthritis	Any	7	Non‐SO	Not serious	Serious	Not serious	Not serious	None	OR 1.52 (1.10 to 2.09)	Very low
Prevalence of cardiovascular conditions	Any	5	Non‐SO	Not serious	Serious	Not serious	Not serious	None	OR 1.92 (1.19 to 3.09)	Very low
Prevalence of diabetes	Any	14	Non‐SO	Not serious	Very serious	Not serious	Not serious	Strong association	OR 2.02 (1.39 to 2.93)	Very low
Prevalence of hyperlipidaemia	Any	5	Non‐SO	Not serious	Serious	Not serious	Not serious	None	OR 1.34 (0.76 to 2.35)	Very low
Prevalence of hypertension	Any	11	Non‐SO	Not serious	Very serious	Not serious	Not serious	None	OR 1.99 (1.34 to 2.97)	Very low
Prevalence of osteoporosis	Any	5	Non‐SO	Not serious	Serious	Not serious	Not serious	None	OR 1.29 (0.79 to 2.11)	Very low
Prevalence of other cardiovascular diseases	Any	11	Non‐SO	Not serious	Serious	Not serious	Not serious	None	OR 1.51 (1.07 to 2.12)	Very low
Lumbar aBMD	Older adults	8	Non‐SO	Not serious	Not serious	Not serious	Not serious	None	MD 0.002 higher (0.03 lower to 0.04 higher)	Low
Prevalence of falls	Older adults	8	Non‐SO	Not serious	Not serious	Not serious	Not serious	None	RR 1.30 (1.10 to 1.54)	Low
Hip aBMD	Older adults	4	Non‐SO	Not serious	Very serious	Not serious	Not serious	None	MD 0.02 lower (0.01 lower to 0.05 higher)	Very low
Femoral neck BMD	Older adults	8	Non‐SO	Not serious	Very serious	Not serious	Not serious	None	MD 0.02 lower (0.08 lower to 0.03 higher)	Very low
Prevalence of non‐vertebral fractures	Older adults	2	Non‐SO	Not serious	Serious	Not serious	Not serious	None	RR 1.32 (0.63 to 2.78)	Very low
Prevalence of falls	Older adults	6	Obese	Not serious	Not serious	Not serious	Not serious	None	RR 1.17 (1.008 to 1.36)	Low
Hip aBMD	Older adults	6	Obese	Not serious	Very serious	Not serious	Not serious	None	MD 0.02 lower (0.05 lower to 0.002 higher)	Very low
Lumbar aBMD	Older adults	11	Obese	Not serious	Serious	Not serious	Not serious	None	MD 0.001 lower (0.02 lower to 0.02 higher)	Very low
Femoral neck BMD	Older adults	8	Obese	Not serious	Very serious	Not serious	Not serious	None	MD 0.02 lower (0.04 to 0.004 lower)	Very low
Prevalence of non‐vertebral fractures	Older adults	2	Obese	Not serious	Serious	Not serious	Not serious	None	RR 1.61 (0.76 to 3.34)	Very low
Lumbar aBMD	Older adults	8	Sarcopenia	Not serious	Not serious	Not serious	Not serious	None	MD 0.03 higher (0.001 to 0.06 higher)	Low
Femoral neck BMD	Older adults	6	Sarcopenia	Not serious	Not serious	Not serious	Not serious	None	MD 0.04 higher (0.006 to 0.07 higher)	Low
Prevalence of falls	Older adults	6	Sarcopenia	Not serious	Not serious	Not serious	Not serious	None	RR 1.07 (0.83 to 1.38)	Low
Prevalence of non‐vertebral fractures	Older adults	2	Sarcopenia	Not serious	Not serious	Not serious	Not serious	None	RR 1.87 (1.08 to 3.24)	Low
Hip aBMD	Older adults	4	Sarcopenia	Not serious	Very serious	Not serious	Not serious	None	MD 0.06 higher (0.01 to 0.11 higher)	Very low

Abbreviations: aBMD, areal bone mineral density; BMD, bone mineral density; CI, confidence interval; MD, mean difference; OR, odds ratio; RR, risk ratio.

**Table 2:** GRADE profile of the associations between sarcopenic obesity (SO) and the outcomes of interest in prospective cohort studies

Outcome	Population	Number of studies	Control group	Risk of bias	Inconsistency	Indirectness	Imprecision	Other considerations	Estimate (95% CI)	Overall certainty of evidence
Incidence of coronary artery disease	Any	2	Non‐SO	Not serious	Not serious	Not serious	Not serious	None	OR 0.99 (0.72 to 1.37)	Low
Incidence of cardiovascular conditions	Any	2	Non‐SO	Not serious	Not serious	Not serious	Not serious	None	OR 1.09 (0.87 to 1.37)	Low
Cardiovascular mortality	Any	3	Non‐SO	Not serious	Very serious	Not serious	Not serious	None	HR 1.63 (1.01 to 2.62)	Very low
Long‐term mortality	Liver transplant	4	Non‐SO	Serious	Not serious	Not serious	Not serious	Strong association	RR 2.08 (1.10 to 3.93)	Low
Short‐term mortality	Liver transplant	4	Non‐SO	Serious	Not serious	Not serious	Not serious	Strong association	RR 2.06 (1.28 to 3.33)	Low
Medium‐term mortality	Liver transplant	4	Non‐SO	Serious	Not serious	Not serious	Not serious	None	RR 1.67 (1.10 to 2.51)	Very low
Recurrence‐free survival	Cancer	3	Non‐SO	Not serious	Not serious	Not serious	Not serious	Strong association	RR 2.28 (1.45 to 3.57)	Moderate
Mortality in pancreatic cancer	Cancer	6	Non‐SO	Not serious	Not serious	Not serious	Not serious	None	RR 1.49 (1.26 to 1.74)	Low
Disease‐free survival	Cancer	2	Non‐SO	Not serious	Not serious	Serious	Not serious	Strong association	RR 2.08 (0.90 to 4.81)	Low
Post‐operative complications	Cancer	7	Non‐SO	Not serious	Not serious	Serious	Not serious	Reporting bias, very strong association	RR 3.08 (2.04 to 5.08)	Low
Mortality	Cancer	13	Non‐SO	Not serious	Serious	Not serious	Not serious	Reporting bias	RR 1.83 (1.41 to 2.38)	Very low

Abbreviations: CI, confidence interval; HR, hazard ratio; OR, odds ratio; RR, risk ratio.

Among cross‐sectional and case–control studies, 30 different outcomes were analysed, among which 18 were statistically significant. Overall, the outcomes included a median of six studies (range: 2–11). Fifteen outcomes were made in any population, and another 15 were among older people. For 20 outcomes, non‐SO was used as control group, while obesity and sarcopenia were used for 5 and 5, respectively. Considering the outcomes involving any population, compared with people without SO, the presence of SO raised the prevalence of cognitive impairment (*k* = 3; OR = 3.46; 95% CI: 2.24–5.32; high certainty of evidence according to the GRADE) and of coronary artery disease (*k* = 2; OR = 2.48; 95% CI: 1.85–3.31) and dyslipidaemia (*k* = 3; OR = 2.50; 95% CI: 1.51–4.15; both moderate certainty of evidence) (*Table* *1*). SO, compared with its absence, was also associated with a low certainty of association with a higher prevalence of functional limitations, lung diseases and stroke. Finally, SO (vs. non‐SO) was associated with a higher prevalence of arthritis, cardiovascular conditions, diabetes and hypertension, even if these associations were supported by a very low certainty of evidence (*Table* *1*).

Taking into account older people, no association was supported by a high or moderate certainty of evidence according to the GRADE, even if SO was associated with a significantly higher proportion of falls compared with non‐SO (*k* = 8; RR = 1.30; 95% CI: 1.10–1.54; low certainty of evidence). In older people, using obese people as a control group, people with SO reported a significantly higher prevalence of falls (RR = 1.17; 95% CI: 1.008–1.36; low certainty of evidence) and lower femoral neck bone mineral density (BMD) values (MD = −0.02 g/cm^2^; 95% CI: −0.04 to 0.004; very low certainty of evidence) (*Table* *1*). Finally, compared with participants with sarcopenia, people with SO reported significantly higher lumbar areal BMD (aBMD) values (MD = 0.03 g/cm^2^; 95% CI: 0.001–0.06), femoral neck BMD (MD = 0.04 g/cm^2^; 95% CI: 0.006–0.07) (low certainty of evidence for both outcomes) and hip aBMD (MD = 0.06 g/cm^2^; 95% CI: 0.01–0.11) (very low certainty of evidence), but a higher prevalence of non‐vertebral fractures (RR = 1.87; 95% CI: 1.08–3.24) (low certainty of evidence).

*Table* *2* summarizes the findings of the prospective cohort studies. In any population, compared with non‐SO, the presence of SO increased the risk of cardiovascular mortality (HR = 1.63; 95% CI: 1.01–2.62; very low certainty of evidence), while no significant association was found with a higher incidence of cardiovascular conditions or coronary artery disease. SO was associated with a higher short‐, medium‐ and long‐term mortality in people undergoing liver transplants (low to very low certainty of evidence) (*Table* *2*). Similarly, in people with cancer, SO was associated with a higher risk of recurrence (*k* = 3; RR = 2.28; 95% CI: 1.45–3.57; moderate certainty of evidence) and a higher mortality rate in pancreatic cancer (*k* = 6; RR = 1.49; 95% CI: 1.26–1.74; low certainty of evidence). Finally, in people with cancer, the presence of SO at baseline increased the risk of post‐operative complications (low certainty of evidence) and mortality (very low certainty of evidence) (*Table* *3*). In reviews summarizing prospective studies, no risk comparison between SO and obesity or sarcopenia was performed.

**Table 3:** Comparison of sarcopenic obesity (SO) with non‐sarcopenic obesity, sarcopenia and obesity

Parameter	Versus non‐SO	Versus obesity	Versus sarcopenia
Lumbar aBMD	Not statistically different LOW	Not statistically different VERY LOW	Significantly higher LOW
Prevalence of falls	Significantly higher prevalence in SO LOW	Significantly higher prevalence in SO LOW	Not statistically different LOW
Hip aBMD	Not statistically different VERY LOW	Not statistically different VERY LOW	Significantly higher VERY LOW
Femoral neck BMD	Not statistically different VERY LOW	Significantly lower in SO VERY LOW	Significantly higher LOW
Prevalence of non‐vertebral fractures	Not statistically different VERY LOW	Not statistically different VERY LOW	Significantly higher LOW

*Note*: The table reports a summary of the comparison between sarcopenic obesity and non‐sarcopenic obesity, obesity or sarcopenia as controls in the only meta‐analysis showing these data.^24^ In capitals, the evidence is reported according to the GRADE. Abbreviations: aBMD, areal bone mineral density; BMD, bone mineral density.

Finally, we retrieved information on the criteria used for the diagnosis of SO in the studies included in the systematic reviews and meta‐analyses identified. Overall, a significant heterogeneity in the criteria used and relative cut‐offs to define obesity, low muscle mass and reduced strength/performance was observed. Besides, the use of muscle function/performance criterion has been scantily considered and reviewed. Specifically on prospective studies (*Table* *4*), which are those enabling to infer a cause‐and‐effect relationship, only three conducted in patients with cancer—and included in two meta‐analyses^24^, ^27^—have considered the combination of the three criteria (obesity + low muscle mass + low muscle strength/physical performance) for the diagnosis of SO. In the general population, three studies have used low handgrip as an exclusive surrogate measure of sarcopenia, and their pooled estimate was significant and without heterogeneity (*I* ^2^ = 0).^22^

**Table 4:** Criteria used for the definition of sarcopenic obesity in prospective studies

Author, year of the systematic review	Number of prospective studies	Setting/condition	Outcomes	Criteria for muscle mass	Criteria for obesity	Criteria for muscle strength	Criteria for physical performance	Combination of muscle mass, obesity, muscle strength or physical performance
Gao, 2022	32	Any cancer	Mortality, recurrence‐free survival, disease‐free survival, surgical complications	SMI (L3) (*n* = 27), SMI (L1) (*n* = 1), BIA (*n* = 2), TAMA (L3) (*n* = 2)	BMI (*n* = 20), VFA (*n* = 7), body fat % (*n* = 2), total BF (*n* = 2), combination of BF and BMI (*n* = 1)	Low HG (*n* = 3)	Low GS (*n* = 3)	Three studies (according to the Asian Working Group for Sarcopenia)
Hegyi, 2019	4	Liver transplant	Mortality	Radiological findings	Not reported	None used	None used	None
Mintziras, 2018	11	Pancreatic cancer	Mortality	SMI (L3) (*n* = 11)	BMI (*n* = 9), total BF (*n* = 2)	None used	None used	None
Wang, 2022	26	Gastrointestinal surgical oncology	Disease‐free survival Major complications Overall complications Overall survival	SMI (L3) (*n* = 17), TAMA (*n* = 7), AWGS criteria (*n* = 2)	BMI (*n* = 8), VFA (*n* = 14), combination of VFA/BMI (*n* = 2), body fat (*n* = 2)	Low HG (*n* = 2)	Low GS (*n* = 2)	Two studies (according to the Asian Working Group for Sarcopenia)
Zhang, 2019	23	Any	Mortality	SMI (*n* = 10), DXA parameters (*n* = 8), others (*n* = 5)	BMI (*n* = 6), BF (*n* = 7), VFA (*n* = 3), WC (*n* = 3), DXA parameters (*n* = 4)	Low HG (*n* = 3)	None	None

Abbreviations: AWGS, Asian Working Group for Sarcopenia; BF, body fat; BIA, bioimpedance analysis; BMI, body mass index; DXA, dual‐energy X‐ray absorptiometry; GS, gait speed; HG, handgrip strength; SMI, skeletal mass index; TAMA, total abdominal muscle area; VFA, visceral fat area; WC, waist circumference.

### Assessment of risk of bias

Using the criteria suggested by the AMSTAR 2, one meta‐analysis was rated as moderate, one critically low and the other seven as low in terms of methodological quality (*Table* *S3*). The most common reasons for potential bias were ‘not clear declaration of the PICO question’ (Question 1), ‘protocol not published before the work’ (Question 2) and ‘missing information regarding the possible role of publication bias in meta‐analyses’ (Question 15).

## Discussion

To the best of our knowledge, this is the first umbrella review trying to comprehensively address the role of SO as a putative risk factor for negative health outcomes. Altogether, we included nine meta‐analyses and approximately 384 710 participants, mainly cross‐sectional and case–control studies, indicating that only a few outcomes (e.g., the prevalence of cognitive impairment, coronary artery disease and dyslipidaemia) are significantly higher compared with the controls (non‐SO). Other statistically significant findings were supported by a low or very low certainty of evidence, indicating that the literature supporting the importance of SO is affected by important biases.

Trying to identify individuals with SO could be a new and important challenge for clinicians all over the world. The European Society for Clinical Nutrition and Metabolism (ESPEN) and the European Association for the Study of Obesity (EASO) tried to reach an expert consensus on a definition and diagnostic criteria for SO^10^ to better characterize this entity and support its identification. First, this consensus affirms that current definitions of obesity and sarcopenia should not be automatically applied to define SO in daily clinical practice, but a step‐by‐step approach should be better.^10^ Besides, in the new proposed algorithm, a considerable and justified weight has been given to muscle function/physical performance, which evaluation should come into the forefront before muscle mass assessment. As confirmed by our umbrella review, this consensus statement encourages the validation of SO as a risk factor in well‐designed prospective follow‐up with the main aim of increasing the scientific evidence needed to identify and treat SO patients.^7^ That is one of the reasons why we collected data and studies for this umbrella review. We believe that our critical and systematic review of all‐type observational studies shows that SO is a highly prevalent condition, but without the concomitant evaluation of muscle strength/performance, its prognostic role is limited. The lack of several significant associations is probably due to the fact that without considering this component as a key factor in defining SO, we probably miss the most important characteristics in terms of prognosis. Therefore, the newer definitions of SO, including a formal assessment of physical performance and muscle strength, could overcome these inherent issues. This would be consistent with the operational algorithm for case finding and confirmation implemented by expert panels for an elderly population,^30^ according to which the evaluation of muscle strength and/or performance should come to the forefront as a more relevant determinant of prognosis.

In this sense, we would like to present an example that could better indicate the state of the literature regarding SO. As mentioned in *Table* *3*, for example, people affected by SO reported similar mean BMD values to participants without this condition but significantly higher lumbar and hip aBMD when compared with people affected by sarcopenia. At the same time, even if SO seems to be associated with a better bone health profile compared with sarcopenic patients, it should be noted that the prevalence of non‐vertebral fractures compared with this population is higher, overall indicating that the research regarding this specific topic probably needs more solid data, particularly from prospective studies, which enables us to infer a cause‐and‐effect relationship more robustly.

The analyses from the cohort studies substantially confirmed the findings of the case–control and cross‐sectional studies in terms of the strength of the association. In fact, SO was associated with a higher incidence of cardiovascular mortality but not with a higher risk of CVDs. Similarly, in people affected by cancer, the presence of SO was associated with a higher risk of recurrence‐free survival compared with non‐SO, with a moderate strength of evidence, but the other statistically significant outcomes were rated as very low to low strength of the association. Therefore, these data confirmed that the prognostic role of SO, particularly in cancer, is promising but still to be confirmed, particularly to evaluate if any risk could arise in comparison with obesity or sarcopenia.

In our opinion, the stratification of the controls in non‐SO, sarcopenia and obesity—as three independent control groups—could better identify the real weight of SO—as a combination of two distinct entities—as both sarcopenia and obesity are associated with a higher risk of negative health outcomes, as shown by other umbrella reviews published in this regard.^3^, ^7^, ^31^ Unfortunately, previous studies and consequent meta‐analyses have scantily addressed a comparison between SO and sarcopenia and obesity alone, thus hampering the evaluation of the real weight of this distinct clinical entity. At the same time, it is noteworthy that often obese older people may also have malnutrition—due to impaired nutritional intake and/or weight loss and/or inflammation—which can lead to sarcopenia.^32^, ^33^ Besides, the independent role of muscle function/performance in refining its diagnosis still needs to be quantified, as we can expect a relevant prognostic impact from its incorporation in the diagnostic workup. This is also supported by the limited subgroup analyses conducted in the meta‐analyses retrieved and included herein or by looking at the few risk estimates of more recent studies using the muscle function/performance domain in the diagnosis; they show more consistent and homogeneous effects on risk.

The findings of our study must be considered with some limitations. First, among the 201 papers initially screened, only nine systematic reviews with meta‐analysis were included, with a small number of studies and mainly case–control and cross‐sectional studies that suffer from some inherent limitations. Second, all the meta‐analyses included contained studies that might significantly differ in design, populations and settings, as well as in terms of the definition of SO, indicating a possible clinical heterogeneity in effects. Finally, the quality of the meta‐analyses was generally low or very low, mainly due to important methodological shortcomings.

In conclusion, SO could be considered a risk factor only for a few conditions (e.g., the prevalence of cognitive impairment, coronary artery disease and dyslipidaemia), with the literature mainly based on cross‐sectional and case–control studies addressing risk estimates compared with non‐SO. Among the outcomes addressed in cohort studies, SO could be considered a poor prognostic factor in cancer, even if the strength of these associations was affected by some biases. Future studies with clear definitions of SO are certainly needed to confirm the importance of SO, particularly when compared with the presence of only sarcopenia or obesity. Furthermore, the relevance of muscle dysfunction/physical performance impairment in its definition still needs to be quantified.

## Funding

None.

## Conflict of interest statement

None.

## Supporting information


**Table S1.** Criteria evidence for the GRADE of the outcomes included.
**Table S2.** List of the excluded references.
**Table S3.** AMSTAR 2 quality assessment of meta‐analyses included.
