# Supplementary material for: Sarcopenic obesity and health outcomes: An umbrella review of systematic reviews with meta‐analysis
Source: J Cachexia Sarcopenia Muscle. 2024 Jun 19;15(4):1264–74. doi: 10.1002/jcsm.13502 (PMC11294015; doi:10.1002/jcsm.13502)
Supplement: Supplementary file 1 — Table S1. Criteria evidence for the GRADE of the outcomes included. Table S2. List of the excluded references. Table S3. AMSTAR 2 quality assessment of meta‐analyses included. [file JCSM-15-1264-s001.docx]

**Supplementary Table 1. Criteria evidence for the GRADE of the outcomes included.**

| **Downgrade** | **Type of study** | **Risk of bias** | **Inconsistency** | **Indirectness** | **Imprecision** | **Publication bias** | **Large effect** |
| --- | --- | --- | --- | --- | --- | --- | --- |
| **-1** | - | Presence of **10-30%** of studies with a high risk of bias according to criteria included in the systematic review | **I^2^ 50-74%** | The question being addressed by the guideline panel is **different** from the available evidence regarding the PICO | The **overall number** of individuals included in studies is low (less than **1000 individuals) OR** (b) large 95% confidence intervals. | **Egger’s test** (p-value) <0.05 | **RR/OR/HR** between 2 and 3 (+1) |
| **-2** | Observational studies | Presence >**30%** of studies with a high risk of bias according to criteria included in the systematic review | **I^2^ > 75%** | The question being addressed by the guideline panel is **markedly different** from the available evidence regarding the PICO | The **overall number** of individuals included in studies is low (less than **1000 individuals AND** (b) large 95% confidence intervals. | - | **RR/OR/HR**  > 3 (+2) |

For **continuous outcomes** “***no effect***” means a SMD with a confidence interval that ***crosses zero***; **appreciable** benefit or appreciable harm means that the **upper or lower confidence limit crosses an effect size of 0.5** in either direction. For **dichotomous outcomes** “***no effect***” means an estimate with a confidence interval that ***crosses one***; **appreciable** benefit or appreciable harm means that the upper or lower confidence limit **crosses a risk of 1.25 or 0.75**.

**Abbreviations: OR:** Odds ratio**; PICO:** Population, Intervention, Comparison and Outcomes; **RR**: Risk Ratio; **HR**: Hazard Ratio

**Supplementary Table 2. List of the excluded references**

| **Meta-analyses of intervention studies (n=11)** | **No meta-analyzable outcomes (n=11)** | **Non sarcopenic obesity (n=5)** | **Doubled (n=5)** | **Only one study (n=2)** | **Protocol (n=1)** |
| --- | --- | --- | --- | --- | --- |
| Ghiotto L, Muollo V, Tatangelo T, Schena F, Rossi AP. Exercise and physical performance in older adults with sarcopenic obesity: A systematic review. *Front Endocrinol (Lausanne)*. 2022;13:913953. doi:10.3389/fendo.2022.913953 | Khadra D, Itani L, Chebaro Y, et al. Association Between Sarcopenic Obesity and Metabolic Syndrome in Adults: A Systematic Review and Meta-Analysis. Curr Cardiol Rev. 2020;16(2):153-162. doi:10.2174/1573403X16666200214104122 | Barnes ME, Elliott JA, McIntyre TV, Boyle EA, Gillis AE, Ridgway PF. Sarcopenia and obesity among patients with soft tissue sarcoma - Association with clinicopathologic characteristics, complications and oncologic outcome: A systematic review and meta-analysis. *Eur J Surg Oncol*. Sep 2021;47(9):2237-2247. doi:10.1016/j.ejso.2021.04.024 | Evans, K., Abdelhafiz, D., & Abdelhafiz, A. H. (2021). Sarcopenic obesity as a determinant of cardiovascular disease risk in older people: a systematic review. Postgraduate medicine, 133(8), 831-842. | O'Connell RM, O'Neill M, MG OR, CB OS, O'Sullivan AW. Sarcopaenia, obesity, sarcopaenic obesity and outcomes following hepatic resection for colorectal liver metastases: a systematic review and meta-analysis. *HPB (Oxford)*. Nov 2022;24(11):1844-1853. doi:10.1016/j.hpb.2022.07.003 | Leahy S, Cassarino M, MD OC, Glynn L, Galvin R. Dynapaenic obesity and its association with health outcomes in older adult populations: protocol for a systematic review. *BMJ Open*. May 24 2019;9(5):e027728. doi:10.1136/bmjopen-2018-027728 |
| Hita-Contreras F, Bueno-Notivol J, Martinez-Amat A, Cruz-Diaz D, Hernandez AV, Perez-Lopez FR. Effect of exercise alone or combined with dietary supplements on anthropometric and physical performance measures in community-dwelling elderly people with sarcopenic obesity: A meta-analysis of randomized controlled trials. *Maturitas*. Oct 2018;116:24-35. doi:10.1016/j.maturitas.2018.07.007 | Batsis JA, Barre LK, Mackenzie TA, Pratt SI, Lopez-Jimenez F, Bartels SJ. Variation in the prevalence of sarcopenia and sarcopenic obesity in older adults associated with different research definitions: dual-energy X-ray absorptiometry data from the National Health and Nutrition Examination Survey 1999-2004. J Am Geriatr Soc. Jun 2013;61(6):974-980. doi:10.1111/jgs.12260 | Merli M, Lattanzi B, Aprile F. Sarcopenic obesity in fatty liver. *Curr Opin Clin Nutr Metab Care*. May 2019;22(3):185-190. doi:10.1097/  MCO.000000000000055 | Silva Neto LS, Medeiros A, Travassos A, OsÓRio NB, Nunes GF. Association between sarcopenic obesity, muscle strength and risk of cardiovascular and cardiometabolic diseases in the elderly: A systematic review. Revista de Nutrição. 2019;32doi:10.1590/1678-9865201932e180237 | Schaap LA, Koster A, Visser M. Adiposity, muscle mass, and muscle strength in relation to functional decline in older persons. *Epidemiol Rev*. 2013;35:51-65. doi:10.1093/epirev/mxs006 |  |
| Liao CD, Tsauo JY, Wu YT, et al. Effects of protein supplementation combined with resistance exercise on body composition and physical function in older adults: a systematic review and meta-analysis. *Am J Clin Nutr*. Oct 2017;106(4):1078-1091. doi:10.3945/ajcn.116.143594 | Corica F, Bianchi G, Corsonello A, Mazzella N, Lattanzio F, Marchesini G. Obesity in the Context of Aging: Quality of Life Considerations. *Pharmacoeconomics*. Jul 2015;33(7):655-72. doi:10.1007/s40273-014-0237-8 | Trevisan C, Crippa A, Ek S, et al. Nutritional Status, Body Mass Index, and the Risk of Falls in Community-Dwelling Older Adults: A Systematic Review and Meta-Analysis. *J Am Med Dir Assoc*. May 2019;20(5):569-582 e7. doi:10.1016/j.jamda.2018.10.027 | Tian S, Xu Y. Association of sarcopenic obesity with the risk of all-cause mortality: A meta-analysis of prospective cohort studies. *Geriatr Gerontol Int*. Feb 2016;16(2):155-66. doi:10.1111/ggi.12579 |  |  |
| Martinez-Amat A, Aibar-Almazan A, Fabrega-Cuadros R, et al. Exercise alone or combined with dietary supplements for sarcopenic obesity in community-dwelling older people: A systematic review of randomized controlled trials. *Maturitas*. Apr 2018;110:92-103. doi:10.1016/j.maturitas.2018.02.005 | Dasarathy J, Rogers K, Rajesh R. Sarcopenia, Sarcopenic Obesity and Frailty; Links to Cognitive Performance in Elders. The American Journal of Geriatric Psychiatry. 2019;27(3):S46-S47. doi:10.1016/j.jagp.2019.01.1998 | Yang JM, Ye H, Zhu Q, et al. Effects of resistance training on body composition and physical function in elderly patients with osteosarcopenic obesity: a systematic review and meta-analysis. *Arch Osteoporos*. Jun 3 2022;17(1):82. doi:10.1007/s11657-022-01120-x | Quan Y, Wang C, Wang L, Li G. Geriatric sarcopenia is associated with hypertension: A systematic review and meta-analysis. J Clin Hypertens (Greenwich). 2023 Sep;25(9):808-816. doi: 10.1111/jch.14714. Epub 2023 Aug 18. PMID: 37594142; PMCID: PMC10497027. |  |  |
| Poggiogalle E, Parrinello E, Barazzoni R, Busetto L, Donini LM. Therapeutic strategies for sarcopenic obesity: a systematic review. *Curr Opin Clin Nutr Metab Care*. Jan 2021;24(1):33-41. doi:10.1097/MCO.0000000000000714 | Donini LM, Poggiogalle E, Migliaccio S, Aversa A, Pinto A. Body composition in sarcopenic obesity: systematic review of the literature. *Mediterranean Journal of Nutrition and Metabolism*. 2013;6(3):191-198. doi:10.1007/s12349-013-0135-1 | Salavatizadeh M, Soltanieh S, Radkhah N, Ataei Kachouei AH, Bahrami A, Khalesi S, Hejazi E. The association between skeletal muscle mass index (SMI) and survival after gastrectomy: A systematic review and meta-analysis of cohort studies. Eur J Surg Oncol. 2023 Nov;49(11):106980. doi: 10.1016/j.ejso.2023.07.006. Epub 2023 Jul 6. PMID: 37451925. | Fang M, Liu C, Liu Y, Tang G, Li C, Guo L. Association between sarcopenia with incident cardio-cerebrovascular disease: A systematic review and meta-analysis. Biosci Trends. 2023 Sep 15;17(4):293-301. doi: 10.5582/bst.2023.01130. Epub 2023 Aug 11. PMID: 37574268. |  |  |
| Poggiogalle E, Migliaccio S, Lenzi A, Donini LM. Treatment of body composition changes in obese and overweight older adults: insight into the phenotype of sarcopenic obesity. *Endocrine*. Dec 2014;47(3):699-716. doi:10.1007/s12020-014-0315-x | Dowling L, Duseja A, Vilaca T, Walsh JS, Goljanek-Whysall K. MicroRNAs in obesity, sarcopenia, and commonalities for sarcopenic obesity: a systematic review. *J Cachexia Sarcopenia Muscle*. Feb 2022;13(1):68-85. doi:10.1002/jcsm.12878 |  |  |  |  |
| Galicia Ernst, I., Torbahn, G., Schwingshackl, L., Knüttel, H., Kob, R., Kemmler, W., ... & Schoene, D. (2022). Outcomes addressed in randomized controlled lifestyle intervention trials in community‐dwelling older people with (sarcopenic) obesity—An evidence map. *Obesity reviews*, *23*(10), e13497. | Gao Q, Mei F, Shang Y, et al. Global prevalence of sarcopenic obesity in older adults: A systematic review and meta-analysis. *Clin Nutr*. Jul 2021;40(7):4633-4641. doi:10.1016/j.clnu.2021.06.009 |  |  |  |  |
| Weinheimer EM, Sands LP, Campbell WW. A systematic review of the separate and combined effects of energy restriction and exercise on fat-free mass in middle-aged and older adults: implications for sarcopenic obesity. *Nutr Rev*. Jul 2010;68(7):375-88. doi:10.1111/j.1753-4887.2010.00298.x | Gortan Cappellari G, Brasacchio C, Laudisio D, et al. Sarcopenic obesity: What about in the cancer setting? *Nutrition*. Jun 2022;98:111624. doi:10.1016/j.nut.2022.111624 |  |  |  |  |
| Yang JM, Luo Y, Zhang JH, et al. Effects of WB-EMS and protein supplementation on body composition, physical function, metabolism and inflammatory biomarkers in middle-aged and elderly patients with sarcopenic obesity: A meta-analysis of randomized controlled trials. *Exp Gerontol*. Sep 2022;166:111886. doi:10.1016/j.exger.2022.111886 | Hanna DJ, Jamieson ST, Lee CS, et al. "Bioelectrical impedance analysis in managing sarcopenic obesity in NAFLD". *Obes Sci Pract*. Oct 2021;7(5):629-645. doi:10.1002/osp4.509 |  |  |  |  |
| Yin YH, Liu JYW, Valimaki M. Effectiveness of non-pharmacological interventions on the management of sarcopenic obesity: A systematic review and meta-analysis. *Exp Gerontol*. Jul 1 2020;135:110937. doi:10.1016/j.exger.2020.110937 | Wijarnpreecha K, Panjawatanan P, Aby E, Ahmed A, Kim D. Nonalcoholic fatty liver disease in the over-60s: Impact of sarcopenia and obesity. *Maturitas*. Jun 2019;124:48-54. doi:10.1016/j.maturitas.2019.03.016 |  |  |  |  |
| Zhuang M, Jin M, Lu T, et al. Effects of three modes of physical activity on physical fitness and hematological parameters in older people with sarcopenic obesity: A systematic review and meta-analysis. *Front Physiol*. 2022;13:917525. doi:10.3389/fphys.2022.917525 | Kovač MB, Pavlin T, Čavka L, Ribnikar D, Spazzapan S, Templeton AJ, Šeruga B. The trajectory of sarcopenia following diagnosis of prostate cancer: A systematic review and meta-analysis. J Geriatr Oncol. 2023 Sep;14(7):101594. doi: 10.1016/j.jgo.2023.101594. Epub 2023 Jul 22. PMID: 37482497. |  |  |  |  |

**Supplementary Table 3. AMSTAR 2 quality assessment of meta-analyses included**

| **Author, Year** | **AMSTAR 2 items ^a, c^** | | | | | | | | | | | | | | | | |
| --- | --- | --- | --- | --- | --- | --- | --- | --- | --- | --- | --- | --- | --- | --- | --- | --- | --- |
|  | **1** | **2 ^b^** | **3** | **4 ^b^** | **5** | **6** | **7 ^b^** | **8** | **9 ^b^** | **10** | **11 ^b^** | **12** | **13 ^b^** | **14** | **15 ^b^** | **16** | **Overall rating** |
| Alves Guimar~aes et al., 2020 | n | n | y | y | y | y | n | n | n | n | / | / | n | n | n | / | LOW |
| Gandham et al., 2020 | n | n | y | y | y | y | n | py | py | n | n | n | n | n | n | n | LOW |
| Gao et al., 2022 | n | n | y | y | y | y | n | py | py | n | n | y | n | n | n | n | LOW |
| Hegyi et al., 2020 | n | n | n | n | n | n | n | n | n | n | n | n | n | n | n | n | CRITICALLY LOW |
| Liu et al., 2022 | n | n | y | py | y | y | n | py | py | n | n | y | n | n | n | n | LOW |
| Mintziras et al., 2018 | n | n | y | y | y | y | n | py | py | n | n | y | y | y | n | n | LOW |
| Wang et al., 2022 | n | n | y | y | y | y | n | py | py | n | n | y | y | y | n | n | LOW |
| Zembura et al., 2022 | n | n | py | py | y | y | n | py | py | n | n | n | n | n | n | n | LOW |
| Zhang et al., 2019 | n | n | y | y | y | y | n | py | py | n | n | y | y | y | n | Y | MODERATE |

Notes:

1. Did the research questions and inclusion criteria for the review include the components of PICO (Population, Intervention, Comparator group, Outcome)? YES/NO. For yes, must have all four.

2. Did the report of the review contain an explicit statement that the review methods were established prior to the conduct of the review and did the report justify any significant deviations from the protocol? YES, PARTIAL YES, NO. For Partial YES: the authors state that they had a written protocol or guide that included ALL the following (review question(s), a search strategy, inclusion/exclusion criteria, a risk of bias assessment). For YES: as for partial yes, plus the protocol should be registered and should also have specified: a meta-analysis/synthesis plan, if appropriate, and a plan for investigating causes of heterogeneity, justification for any deviations from the protocol.

3. Did the review authors explain their selection of the study designs for inclusion in the review? YES/NO. For YES, the review should satisfy one of the following: explanation for including only RCTs, or explanation for including only NRSI, or explanation for including both RCTs and NRSI.

4. Did the review authors use a comprehensive literature search strategy? YES, PARTIAL YES, NO. for PARTIAL YES must have all of the following: searched at least 2 databases (relevant to research question), provided key word and/or search strategy, justified publication restrictions (eg. Language). For YES should also have all of the following: searched the reference lists/biographies of included studies, searched trial/study registries, included/consulted content experts in the field, searched for grey literature where relevant, conducted search within 24 months of completion of the review.

5. Did the review authors perform study selection in duplicate? YES/NO. for YES, either ONE of the following: at least two reviewers independently agreed on selection of eligible studies and achieved consensus on which studies to include OR two reviewers selected a sample of eligible studies and achieved good agreement (at least 80 per cent) with the remainder selected by one reviewer.

6. Did the review authors perform data extraction in duplicate? YES/NO. For YES, either one of the following: at least two reviewers achieved consensus on which data to extract from included studies OR two reviewers extracted data from a sample of eligible studies and achieved good agreement (at least 80 per cent) with the remainder extracted by one reviewer.

7. Did the review authors provide a list of excluded studies to justify the exclusions? YES, PARTIAL YES, NO. FOR partial yes must provide a list of all potentially relevant studies that were read in full text form but excluded from the review. For YES must also have justified the exclusion from the review of each potentially relevant study.

8. Did the review authors describe the included studies in adequate detail? YES, PARTIAL YES, NO. For PARTIAL YES, must describe all of the following: populations, interventions, comparators, outcomes, research designs. For YES should also have all of the following: described populations in detail, described intervention and comparator in detail (including doses where relevant), described study setting, timeframe or follow-up.

9. Did the review authors use a satisfactory technique for assessing the risk of bias (RoB) in individual studies that were included in the review? For RCTs: YES, PARTIAL YES, NO, INCLUDES ONLY NRSI. For PARTIAL YES must have assessed RoB from unconcealed allocation and lack of blinding of patients and assessors when assessing outcomes (unnecessary for objective outcomes such as all cause mortality); for YES must also have assessed RoB from allocation sequence that was not truly random and selection of the reported result from among multiple measurements or analyses of a specified outcome. For NRSI (Non Randomized Studies of Intervention): YES, PARTIAL YES, NO, INCLUDES ONLY RCTs. For PARTIAL YES must have assessed RoB from confounding and from selection bias. For YES, must also have assessed methods used to ascertain exposures and outcomes, and selection of the reported results from among multiple measurements or analyses of a specified outcome.

10. Did the review authors report on the sources of funding for the studies included in the review? YES/NO. For YES: must have reported on the sources of funding for individual studies included in the review. Note: reporting that the reviewers looked for this information but it was not reported by study authors also qualifies

11. If meta-analysis was performed, did the review authors use appropriate methods for statistical combination of results? For RCTs: YES, NO, NO META-ANALYSIS. For YES: the authors justified combining the data in a meta-analysis and they used an appropriate weighted technique to combine study results and adjusted for heterogeneity if present and investigated the causes of heterogeneity. For NRSI: YES, NO, NO META-ANALYSIS CONDUCTED. For YES: the authors justified combining the data in a meta-analysis and they used an appropriate weighted technique to combine study results, adjusting for heterogeneity if present, and they statistically combined effects estimates from NRSI that were adjusted for confounding, rather than combining raw data, or justified combining raw data when adjusted effect estimates were not available, and they reported separate summary estimates for RCTs and NRSI separately when both were included in the review.

12. If meta-analysis was performed, did the review authors assess the potential impact of RoB in individual studies on the results of the meta-analysis or other evidence synthesis? YES, NO, NO META-ANALYSIS INCLUDED. For YES: included only low risk of bias RCTs or, if the pooled estimate was based on RCTs and/or NRSI at variable RoB, the authors performed analysis ton investigate possible impact of RoB on summary estimates of effect.

13. Did the review authors account for RoB in individual studies when interpreting/discussing the results of the review? YES/NO. for YES: included only low risk of bias RCTs or, if RCTs with moderate or high RoB, or NRSI were included, the review provided a discussion of the key impact of RoB on the results

14. Did the review authors provide a satisfactory explanation for, and discussion of, any heterogeneity observed in the results of the review? YES/NO. For Yes: there was no significant heterogeneity in the results OR if heterogeneity was present the authors performed an investigation of sources of any heterogeneity in the results and discussed the impact of this on the results of the review

15. If they performed quantitative synthesis did the review authors carry out an adequate investigation of publication bias (small study bias) and discuss its likely impact on the results of the review? YES, NO, NO META-ANALYSIS CONDUCTED. For YES: performed graphical statistical tests for publication bias and discussed the likelihood and magnitude of impact of publication bias

16. Did the review authors report any potential sources of conflict of interest, including any funding they received for conducting the review? YES/NO. For Yes: the authors reported no competing interests OR the authors described their funding sources and how they managed potential conflicts of interest.

d Rating overall confidence in the results of the review:

HIGH: no on one non-critical weakness: the systematic review provides an accurate and comprehensive summary of the results of the available studies that address the question of interest

MODERATE: more than one non critical weakness (multiple non-critical weaknesses may diminish confidence in the review and it may be appropriate to move the overall appraisal down from moderate to low confidence): the systematic review has more than one weakness but no critical flaws. It may provide an accurate summary of the results of the available studies that were included in the review

LOW: one critical flaw with or without non-critical weaknesses: the review has a critical flaw and may not provide an accurate and comprehensive summary of the available studies that address the question of interest

CRITICALLY LOW: more than one critical flaw with or without non-critical weaknesses: the review has more than one critical flaw and should not be relied on to provide an accurate and comprehensive summary of the available studies.
